# Monitoring of Chlorogenic Acid and Antioxidant Capacity of *Solanum melongena* L. (Eggplant) under Different Heat and Storage Treatments

**DOI:** 10.3390/antiox8070234

**Published:** 2019-07-20

**Authors:** Petra Šilarová, Lila Boulekbache-Makhlouf, Federica Pellati, Lenka Česlová

**Affiliations:** 1 Department of Analytical Chemistry, Faculty of Chemical Technology, University of Pardubice, Studentská 573, CZ-53210 Pardubice, Czech Republic; 2 Department of Inorganic Technology, Faculty of Chemical Technology, University of Pardubice, Doubravice 41, CZ-53210 Pardubice, Czech Republic; 3 Laboratoire de Biomathématiques, Biophysique, Biochimie, et Scientométrie (L3BS), Faculté des Sciences de la Nature et de la Vie, Université de Bejaia, Bejaia 06000, Algeria; 4 Department of Life Sciences, University of Modena and Reggio Emilia, Via G. Campi 103, IT-41125 Modena, Italy

**Keywords:** *Solanum melongena* L., eggplant, cooking, HPLC, chlorogenic acid, antioxidant activity

## Abstract

*Solanum melongena* L., also known as eggplant, is a widely consumed vegetable and it is well-known for its beneficial antioxidant properties, due to phenolic compounds. In this work, the influence of different cooking procedures on the content of chlorogenic acid was evaluated on eggplant samples of different geographic origin by high-performance liquid chromatography (HPLC). An easy and quick extraction procedure with 50% methanol as the extraction solvent was optimized for the first time by means of a design-of-experiment and applied to heat treated samples of eggplant. The antioxidant capacity of eggplant extracts was also evaluated by using the ABTS assay and it was correlated with the data obtained by the HPLC method. The content of chlorogenic acid was different in each heat-treated eggplant sample and it depended on the temperature applied during the cooking procedure. In particular, an increase of chlorogenic acid content with rising temperature was observed. Conversely, a very high temperature (250 °C) caused a decrease of chlorogenic acid amount. The influence of storage on the content of chlorogenic acid was also monitored. While the level of chlorogenic acid in fresh samples decreased during four weeks of storage, an increase in its content in heat treated eggplant was observed within the same period. Multivariate data analysis was used to classify eggplant samples into different groups, according to the country of origin and heat treatment procedure. This study provides new insights to preserve the antioxidant properties of eggplant phenolics during different thermal and storage treatments in order to highlight their health promoting effects.

## 1. Introduction

*Solanum melongena* L., also known as eggplant, is a native plant from Southeast Asia that was domesticated more than 4000 years ago. Its color, size and shape significantly depend on the specific variety [1]. The world production of eggplant in 2017 was around 52.3 million tons, with China being the main producer, followed by India, Egypt, Turkey and Iran. The production of eggplant in Europe in the same year was around 0.93 million tons, Italy being the main producer, followed by Spain, Romania, Ukraine and Greece [2].

Even if eggplant is usually considered to be a vegetable, it is a fruit from the botanical point of view. Eggplant is known for the antioxidant activity related to its phenolic compounds [3], with chlorogenic acid (5-*O*-caffeoylquinic acid) being the most abundant one [4,5]. Isomers of chlorogenic acid, such as cryptochlorogenic acid (4-*O*-caffeoylquinic acid) and neochlorogenic acid (3-*O*-caffenoylquinic acid), are minor eggplant phenolics [6,7]. A high amount of chlorogenic acid derivatives has been found in vegetables related to eggplant, such as cardoon, with a content around 5 mg/g [8]. On the other hand, chlorogenic acid has not been detected in green pepper [8] and fresh celery roots [9]. A very low amount of this compound has been determined in cucumber [10].

In addition to the antioxidant activity, chlorogenic acid and related polyphenols have many biological activities, including the anti-inflammatory, antimutagenic and antiproliferative ones [11]. These compounds can also influence the activity of trypsin, amylase and other enzymes [12,13]. Some studies have been specifically focused on the comparison of the antioxidant capacity of different varieties of eggplant [1,14].

It is well-known that heat treatment before consumption significantly affects vegetable composition, by influencing both the polyphenol profile and the antioxidant capacity. In most cases, heating causes a decrease of phenolic compounds, but, sometimes, the concentration of phenolics may increase in comparison to the fresh plant material [15,16,17]. The effect of the cooking technique depends on the polarity of the medium where the process is carried out: indeed, hydrothermal processes have a negative effect on the content of soluble antioxidants (including phenolic substances) [18], while non-polar media, which are used during frying process, do not cause a dramatic decrease of phenolic compounds [18]. The decrease of the amount of phenolics during cooking also depends on the ratio between water and vegetable, cooking time and surface size [19]. The degradation kinetics of several compounds have been examined in different fruit and vegetables, including beetroot, green pea, eggplant, green pepper, apple, cranberries, sweet corn and murta berries [20,21,22,23,24]. The knowledge of the degradation kinetics is important during thermal processing to predict the decrease or increase of the content of phenolics or other nutrients.

Conventional extraction techniques for polyphenolic compounds from food samples are carried out with solid–liquid extraction procedures by using mixtures of organic solvents, such as ethanol, methanol or acetone and water [25,26,27,28,29]. The analysis of chlorogenic acid is usually performed by reversed-phase high-performance liquid chromatography (RP-HPLC) with an octadecyl silica gel (C_18_) stationary phase and aqueous methanol or acetonitrile as the mobile phase [30,31]. Detection is traditionally carried out by using a spectrophotometric detector or by mass spectrometry (MS) [4,32,33,34,35].

In the light of all the above, this study was aimed at the assessment of the influence of different temperatures and cooking techniques on eggplant phenolics, by monitoring both the content of its main compound (chlorogenic acid) by means of HPLC and the antioxidant activity of the final product. The extraction of chlorogenic acid from eggplant samples was optimized by using a design-of-experiment approach. The HPLC method allowed us to check also other compounds arising after thermal processing of eggplant.

## 2. Materials and Methods

### 2.1. Chemicals and Solvents

Two eggplant samples from the Netherlands and Spain were purchased from local markets (Pardubice, Czech Republic). Another sample was obtained from a private grower (Čepí, Czech Republic) and two samples of eggplant were from Northern and Southern Italy (Modena and Messina, respectively). All these samples were purple, oval shape with intermediate size, belonging to the American varietal group [36,37]. The samples were immediately analyzed and, for the evaluation of the effect of storage, the samples were kept at low temperature (4 °C).

The standards of chlorogenic acid (i.e., 5-*O*-caffeoylquinic acid) and Trolox (TE) were purchased from Sigma-Aldrich (Prague, Czech Republic). Potassium peroxodisulphate (Lach-Ner, Neratovice, Czech Republic) and 2,2’-azino-bis(3-ethylbenzothiazoline-6-sulfonic acid) diammonium salt (ABTS) from Sigma-Aldrich (USA) were used to measure the antioxidant activity.

Chromatographic grade methanol, acetone, acetonitrile and formic acid were from Sigma-Aldrich (USA). Deionized water was prepared by Milli-Q purification system (Merck Millipore, Germany).

### 2.2. Extraction of Chlorogenic Acid from Eggplant

The eggplant samples were sliced (3 g) and three pieces of the same sample were heat treated (roasted, baked, grilled) for 5 min. Roasting was carried out on a pan from natural stone without oil addition (*T* = 150 °C). Baking was performed in a hot air oven at 250 °C and contact grill (*T* = 190 °C) was used for grilling of the samples.

The optimization of chlorogenic acid extraction was performed by using a design-of-experiment approach (Box, Hunter and Hunter, 2^k-p^ model) [38]. Three variables were selected, namely the type of solvent (acetone or methanol), the concentration of solvent in water (50–70%, *v*/*v*) and the addition of formic acid (0–0.5%, *v*/*v*). The fresh, roasted, grilled and baked samples were chopped and extracted for three minutes in 40 mL of solvent. After that, 3 mL of the extract were evaporated to dryness under nitrogen and the residue was dissolved in 1 mL of 50% methanol. Finally, the extract was filtered by using a PTFE (0.45 µm) syringe filter and injected into the HPLC system. The experiments were performed in triplicate for each eggplant sample.

### 2.3. HPLC Analysis of Chlorogenic Acid

The HPLC system was equipped with a LC-20AD binary gradient pump, a DGU-20A degassing unit, a LCO 102 Single thermostat column (Ecom, Prague, Czech Republic), a 7725i manual dosing valve with 2 μL loop (Rheodyne, Rohnert Park, CA, USA) and a SPD M30A spectrophotometric detector (Shimadzu, Kyoto, Japan). For the identification of unknown compounds, the mass spectrometer QTRAP 4500 (AB Sciex, Redwood City, CA, USA) with electrospray (ESI) and atmospheric pressure chemical ionization (APCI) was employed. The optimal separation was achieved on an Ascentis Express C_18_ column (150 × 3.0 mm i.d., 2.7 μm) at 30 °C, with a gradient elution of the mobile phase consisting of 0.3% formic acid in water (A) and acetonitrile (B). The gradient profile was as follows: 0–5 min—10% B; 5–6 min—85% B. The flow rate was 0.5 mL/min and the injection volume was 2 μL. The conditions for electrospray ionisation (ESI) analysis operated in both the negative and positive-ion mode were: curtain gas (20 psi), temperature (500 °C), ion source gas 1 (50 psi) and ion source gas 2 (50 psi). The conditions for atmospheric pressure chemical ionization (APCI)-MS analysis, operated both in the negative and in the positive ion mode, were the following: curtain gas (20 psi), temperature (400 °C), ion source gas 1 (30 psi) and ion source gas 2 (50 psi). The entrance potential was 10 V and the declustering potential was changed to enhance the sensitivity of the detection from 30 to 120 V in case of both ionization techniques.

### 2.4. Determination of the Antioxidant Activity of Eggplant Samples

The antioxidant activity of eggplant extracts was measured by using a UV-2450 spectrophotometer (Shimadzu, Kyoto, Japan). The preparation of the ABTS radical cation was adapted from the literature with slight modifications [39]. Briefly, ABTS was dissolved in deionized water and mixed with potassium peroxodisulphate. The solution was left for 12–16 h in the dark and then it was diluted to achieve an absorbance value of about 0.8. The extract from fresh and heat-treated eggplants was mixed with this working solution and the decrease of absorbance after 10 min of reaction (in the dark) was monitored at a wavelength of 734 nm. The antioxidant activity was expressed as Trolox equivalent antioxidant capacity (TEAC). The calibration solutions were prepared by dilution of Trolox in methanol (*c* = 0.01 mol/L) in the range of 0.15–1.15 µmol/L. The calibration data were measured at ten concentration levels and each level was repeated five times (*n* = 5). To determine the antioxidant activity, the fresh sample and heat-treated samples (roasted, baked and grilled) were measured in five repetitions. Then, the TEAC value was referred to 1 g fresh weight (FW).

### 2.5. Statistical Analysis

The least square linear regression was applied for the fitting of all calibration data (QCEXPERT 2.9, TriloByte, Medford, OR, USA). The graphical diagnostics (Pregibon, Williams and L-R graphs, Hoboken, NJ, USA) were used to identify influential points. STATISTICA 12 (StatSoft, Inc., Tulsa, OK, USA) was used for multivariate data analysis. All statistical tests were carried out at a significance level of 95% (*α* = 0.05).

## 3. Results and Discussions

### 3.1. Optimization of Eggplant Extraction Conditions

**Figure 1:**
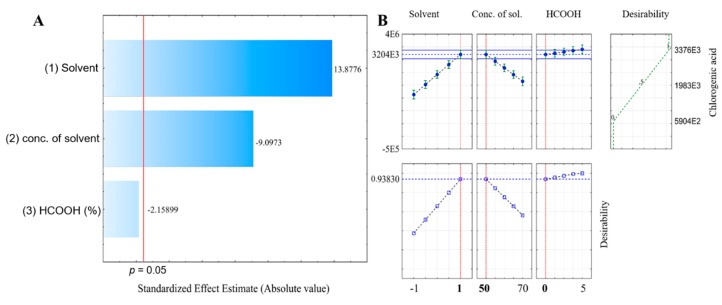
The Pareto graph of the standardized effects in the factorial design for three variables (**A**) and the profiles of predicted values and desirability function evaluated for chlorogenic acid peak area (**B**). Dashed line indicates optimal values (STATISTICA, StatSoft).

The suitable procedure for the extraction of chlorogenic acid from eggplant samples was optimized by using a design-of-experiment approach. The type of solvent (acetone or methanol), its concentration (50–70% in water, *v*/*v*) and formic acid addition (0–0.5%, *v*/*v*) were the optimized parameters. The dependent variable for the evaluation of the extraction process was the peak area of chlorogenic acid. The type of organic solvent and the concentration were significant for the extraction process, whereas formic acid addition was under the level of significance in the Pareto graph (Figure 1A). The highest extraction efficiency of chlorogenic acid was achieved by using 50% (*v*/*v*) methanol, as shown in the profiles of predicted values and desirability (Figure 1B).

### 3.2. Optimization of the HPLC Conditions

**Figure 2:**
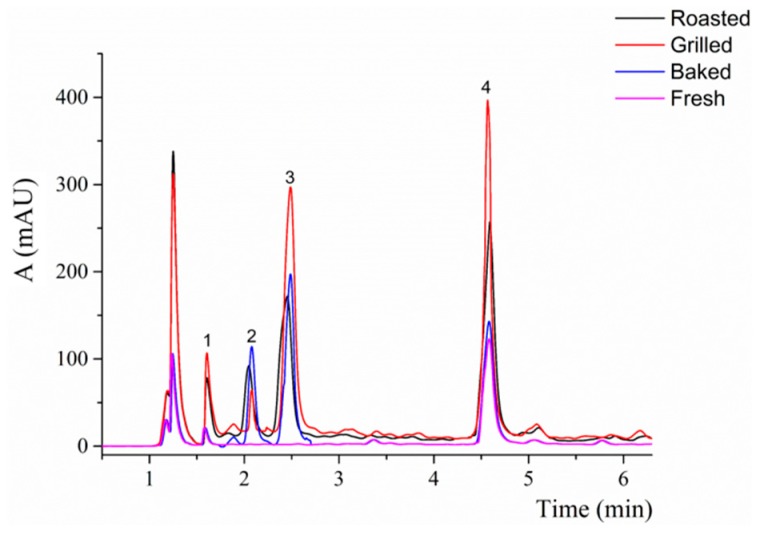
Optimized chromatographic separation of chlorogenic acid and other compounds before and after thermal processing of eggplant. 1—(dihydrocoumaroyl glucoside) amide isomer, 2—*N*-caffeoylputrescine isomer, 3—5-hydroxymethylfurfural, 4—chlorogenic acid.

A core-shell (or fused-core) C_18_ column (150 × 3.0 mm i.d., 2.7 μm) was used for the analysis of chlorogenic acid in the extracts obtained from eggplant samples. The optimization of the chromatographic separation included these parameters: the mobile phase composition (acetonitrile or methanol), the concentration of formic acid, the gradient profile and the column temperature (30–40 °C). The optimized separation is shown in Figure 2, where the differences between a fresh and a heat-treated eggplant sample are shown. The three polar compounds eluting at the beginning of the chromatogram (peaks number 1–3, Figure 2) were monitored during the heat treatment of eggplant samples. Their UV spectra, together with the ESI and APCI mass spectra recorded both in the negative and in the positive-ion mode, were used for the identification of these compounds.

### 3.3. HPLC Method Validation

Eight calibration solutions of chlorogenic acid were prepared by a sequential dilution of the stock methanol solution (*c* = 1 g/L) with the mobile phase at the initial gradient composition (10% methanol). The individual calibration solutions were analyzed three times (*n* = 3). The good linearity of the calibration curve was demonstrated by the coefficient of determination (*r^2^* = 0.9991). The Student’s *t*-test was used for the examination of the significance of the intercept of regression straight-lines (QC Expert 2.9, Trilobyte, Pardubice, Czech Republic). The limit of detection (LOD), calculated as the concentration yielding signal-to-noise ratio (S/N) of 3, was 7 μg/L; the limit of quantification (LOQ), corresponding to a S/N ratio of 10, was 23 μg/L. Two calibration solutions were analyzed ten times to verify the accuracy and the precision of the method. The percent relative standard deviation (%RSD) of the peak areas and retention times did not exceed 4%. The intra-day repeatability was tested by analyzing ten extracts prepared from grilled eggplant samples in one day, using the optimized HPLC method. The inter-day repeatability was checked two days later in the same way (10 extracts from the same sample). The %RSD was below 6% for the intra-day precision and below 11% for the inter-day precision, thus confirming the satisfactory robustness and repeatability of the developed extraction process and HPLC method.

### 3.4. Antioxidant Activity of Eggplant Samples under Different Cooking Conditions

**Figure 3:**
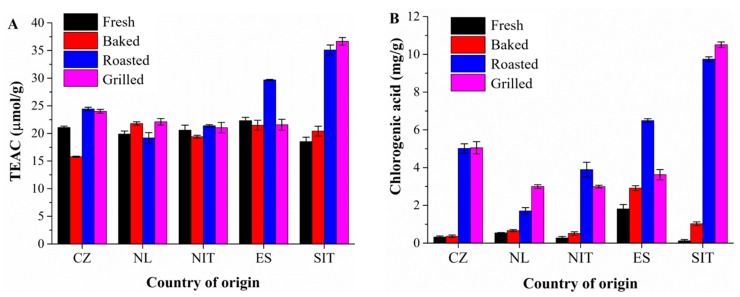
Trolox equivalent antioxidant capacity (TEAC) values (µmol/g) (**A**) and content of chlorogenic acid (mg/g, fresh weight (FW)) in eggplant samples measured by HPLC (**B**). CZ—Czech Republic, NL—the Netherlands, NIT—Northern Italy, ES—Spain, SIT—Southern Italy.

The values of the antioxidant capacity of the eggplant samples are shown in Figure 3A. Data were in the range of 18.5–22.3 µmol TE/g FW for fresh samples, 15.7–21.4 µmol TE/g FW for baked samples, 19.2–35.1 µmol TE/g FW for roasted samples and 21.1–36.6 µmol TE/g FW for grilled samples. The highest value of TEAC was obtained in grilled samples from Southern Italy and the lowest one was determined in the baked sample from the Czech Republic (Figure 3A). The values of the antioxidant capacity of heat-treated samples increased as a function of temperature. Another study carried out by Chumyam et al. [40] has described similar results. In particular, the authors have found that the antioxidant capacity of heat-treated eggplant fruit by means of the ABTS method was dependent on the heat treatment and on the temperature of the cooking procedure, and the antioxidant capacity increased by increasing temperature [40]. Similar findings have been obtained in the study of Ramírez-Anaya et al. [25], who have determined the antioxidant capacity of eggplant prepared by four cooking techniques by using the ABTS method. Later, Arkoub-Djermoune et al. [17] have demonstrated that both eggplant health-promoting compounds and ABTS antioxidant activity are significantly affected by thermal procedures, with oven cooking being the better way to enhance the antioxidant properties of eggplant.

The effects of different cooking methods on the antioxidant capacity has also been previously investigated in artichoke samples, where the major phenolic compounds are 5-*O*-caffeoylquinic and 1,5-di-*O*-caffeoylquinic acids [41]. After the cooking treatment, an increase of the overall caffeoylquinic acid concentration was observed, due to the formation of different dicaffeoylquinic acid isomers, particularly in steamed and fried samples.

The impact of the cooking treatment on the antioxidant capacity of vegetables has also been evaluated by means of the DPPH method in onion, green pepper and cardoon [8]. All heat treatments led to an increased concentration of phenolic compounds and an enhanced antioxidant capacity [8].

### 3.5. Effect of Thermal Processing on Eggplant Phenolics

The major compound observed in the extracts prepared from fresh and cooked eggplant samples was chlorogenic acid (Figure 2), in agreement with the results previously described in the literature [29,42,43,44], where this component represents 50–96% of all phenolic compounds. In our study, the content of chlorogenic acid in fresh samples was in the range 0.1–1.9 mg/g and it increased during the heat treatments of the samples (Figure 3B). Among the fresh samples, the amount of chlorogenic acid in eggplant from Spain was six times higher than that found in the other samples (1.86 ± 0.23 mg/g, FW). Similar results have been previously described in the literature for the level of chlorogenic acid in fresh eggplant (0.5–2.0 mg/g). As for eggplant dried samples, the amount of chlorogenic acid described in the literature varies from 0.5 to 13.0 mg/g [29,42,43,44]. The content of chlorogenic acid is known to be highly dependent on the cultivar and on the harvesting conditions [29].

In addition to chlorogenic acid, other polar compounds were detected in the samples analyzed after the cooking procedures. HPLC-MS was used to identify these compounds. Based on the ESI and APCI mass spectra recorded both in the positive and in the negative-ion mode, the main peaks observed after thermal treatment of the samples (peaks number 1–3, Figure 2) were associated to (dihydrocoumaroyl glucoside) amide isomer, *N*-caffeoylputrescine isomer and 5-hydroxymethylfurfural. The identification was supported by UV spectra recorded in the range 200–600 nm. These findings are in agreement with other studies [45,46,47]. APCI was necessary, due to the low ionization efficiency of 5-hydroxymethylfurfural with ESI [48]. Besides these three main compounds, phenylalanine and other isomers of (dihydrocoumaroyl glucoside) amide and *N*-caffeoylputrescine were detected in lower extent. In other studies, hydroxytyrosol and tyrosol [25] or other derivatives of putrescine [46] have been found after different heat treatments of eggplant. However, those compounds were not detected in the present study.

During the eggplant cooking process, the content of extractable chlorogenic acid increased, as shown in Figure 3B. A significant increase of chlorogenic acid content was achieved by grilling and roasting in comparison with the fresh material. A similar phenomenon has been observed in the study of Ramírez-Anaya et al. [25], who have observed that the content of chlorogenic acid increased ten times during frying and four times during boiling. Together with the increasing level of chlorogenic acid, other compounds (e.g., Maillard reaction products) are formed during the heat treatment of eggplant [25]. Maillard reaction products in heated food have been reported to possess antioxidant activity and even pro-oxidant properties [49]. The increased amount of chlorogenic acid found in heated eggplant samples is probably caused by isomerization and hydrolysis reactions, leading to a substantial redistribution of phenolic acid concentration, due to the massive trans-esterification phenomenon occurring during its processing [41]. As observed by other authors, this is particularly evident for 3,5-and 4,5-di-*O*-caffeoylquinic acids, which have very low concentrations in the fresh product and they are extensively produced during processing [41]. A further possible explanation of this phenomenon is the thermal destruction of cell walls and sub-cellular compartments during the cooking process that favors the release of the compounds [8]. Higher amounts of phenolic compounds in griddled samples of onion, pepper and cardoon, with increments of 57%, 26% and 203% compared to the fresh material, have also been previously reported [8].

**Figure 4:**
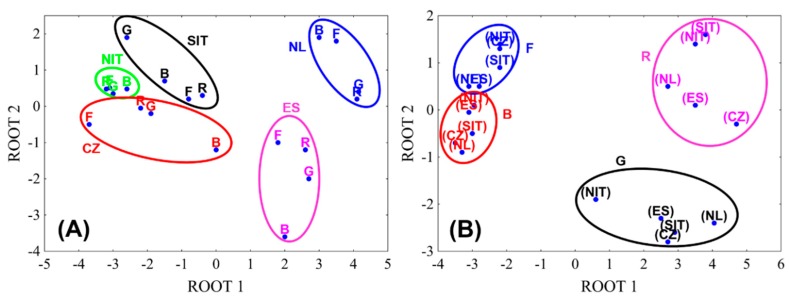
Scatterplots of the linear discriminant scores providing a visual impression of how well the Fisher linear discriminant functions classify the data: (**A**) according to the country of origin (STATISTICA, StatSoft); (**B**) according to the cooking procedure (STATISTICA, StatSoft). F—fresh, B—baked, R—roasted, G—grilled, CZ—Czech Republic, NL—The Netherlands, NIT—Northern Italy, ES—Spain, SIT—Southern Italy.

A discriminant analysis was used to classify eggplant samples. The eggplant samples were marked according with the thermal cooking process (F—fresh, B—baked, R—roasted, G—grilled) and the country of origin (CZ—Czech Republic, NL—the Netherlands, NIT—Northern Italy, ES—Spain, SIT—Southern Italy). Figure 4A shows the eggplant samples divided into different groups, according to their country of origin. The samples from Southern Italy (SIT), Northern Italy (NIT) and Czech Republic (CZ) are close to each other in the graph and this indicated that they were similar in their chemical composition. On the other hand, eggplant samples from Spain (ES) and the Netherlands (NL) are located far from the others, indicating a different composition. Eggplant samples were purchased in local markets and it is not possible to know the conditions of cultivation, which can influence the chemical composition. Furthermore, humidity and temperature play a role during the transport of vegetables and fruits [50]. The recommended storage and transportation conditions for eggplant are 10 °C and 85–95% humidity. In addition, many crops are often harvested before ripening and they are stored in modified atmospheres and under the influence of gases that slow down the ripening process.

By means of the discriminant analysis, eggplant samples were divided into four groups, according to the thermal cooking process (Figure 4B). The fresh (group F) and baked (~250 °C, group B) samples are close in the graph, unlike the roasted (~150 °C, group R) and grilled (~190 °C, group G) ones. It is evident that the content of phenolics increased with increasing temperature. However, the high temperature reached during baking caused the degradation of chlorogenic acid. This effect has also been observed by del Castillo et al. in roasted coffee beans [51], where an increase of chlorogenic acid content was observed by increasing temperature to further decrease in dark roasted samples processed at 240 °C.

### 3.6. Effect of Storage on Eggplant Phenolics

The eggplant samples from Spain were stored in the fridge at ~4 °C for four weeks. Every week, the different cooking treatments were repeated, followed by the extraction and HPLC analysis to quantify chlorogenic acid.

**Figure 5:**
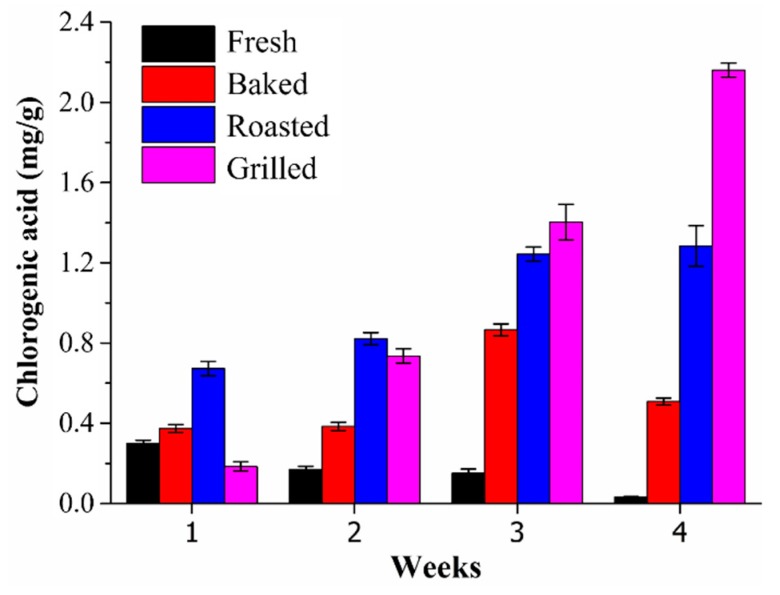
Effect of storage time on chlorogenic acid content (mg/g, FW) in the eggplant sample from Spain after different cooking procedures.

The amount of chlorogenic acid decreased in fresh eggplants from Spain during the four weeks of storage (Figure 5), in agreement with the literature [52]. Galani et al. [53] have observed an increase of chlorogenic acid in dill leaves during storage in the fridge, which was paralleled by a decrease in total phenolics content and antioxidant capacity. In the present study, an important decrease in the level of chlorogenic acid was observed in the first week of storage: indeed, the content of this compound in fresh eggplants (0.30 mg/g, FW) was reduced by six times in comparison with the samples measured at the beginning of the study (1.86 mg/g, FW). However, an increase of chlorogenic acid in heat treated eggplant samples during the four weeks of storage was produced (Figure 5), in particular for grilled samples (2.16 mg/g vs. 0.19 mg/g, FW).

## 4. Conclusions

The influence of different cooking procedures on chlorogenic acid content in eggplant samples of different geographic origin was evaluated by using a quick and easy extraction method, which was optimized here for the first time, followed by HPLC analysis. An increase of chlorogenic acid content with rising cooking temperature was observed as well as those of other polar compounds eluting at the beginning of the HPLC separation. Conversely, a very high temperature (250 °C) caused a decrease in the amount of this phenolic compound. The results of the antioxidant capacity as measured by the ABTS method showed good correlation with chlorogenic acid levels determined by HPLC.

The discriminant analysis of the results allowed us to divide the samples into different groups, according to the country of origin and also to the cooking process. As for chlorogenic acid content, grilling, roasting and baking can be recommended for the domestic cooking of eggplant, with grilling providing the highest levels. The influence of storage on the content of chlorogenic acid was also monitored, observing a decrease within four weeks in fresh eggplant samples, but an increase was observed in heat treated samples over time.
